# High-Porosity Foam-Based Iontronic Pressure Sensor with Superhigh Sensitivity of 9280 kPa^−1^

**DOI:** 10.1007/s40820-021-00770-9

**Published:** 2021-12-09

**Authors:** Qingxian Liu, Yuan Liu, Junli Shi, Zhiguang Liu, Quan Wang, Chuan Fei Guo

**Affiliations:** 1 School of Astronautics, Harbin Institute of Technology, Harbin, 150001 Heilongjiang People’s Republic of China; 2 Department of Materials Science and Engineering, Southern University of Science and Technology, Shenzhen, 518055 People’s Republic of China; 3 Department of Mechanics and Aerospace Engineering, Southern University of Science and Technology, Shenzhen, 518055 Guangdong People’s Republic of China; 4 Department of Civil and Environmental Engineering, Shantou University, Shantou, 515063 Guangdong People’s Republic of China; 5 Department of Mechanical Engineering, Massachusetts Institute of Technology, Cambridge, MA 02139 USA; 6 Department of Physics and TcSUH, University of Houston, Houston, TX 77204 USA; 7 Guangdong Provincial Key Laboratory of Functional Oxide Materials and Devices, Southern University of Science and Technology, Shenzhen, 518055 Guangdong People’s Republic of China; 8 Present Address: 320 Crescent Village Circle Unit 1413, San Jose, CA 95134 USA

**Keywords:** High porosity, Elastic modulus, Compression deformation, Iontronic pressure sensor, Superhigh sensitivity

## Abstract

Flexible pressure sensors with high sensitivity are desired in the fields of electronic skins, human–machine interfaces, and health monitoring. Employing ionic soft materials with microstructured architectures in the functional layer is an effective way that can enhance the amplitude of capacitance signal due to generated electron double layer and thus improve the sensitivity of capacitive-type pressure sensors. However, the requirement of specific apparatus and the complex fabrication process to build such microstructures lead to high cost and low productivity. Here, we report a simple strategy that uses open-cell polyurethane foams with high porosity as a continuous three-dimensional network skeleton to load with ionic liquid in a one-step soak process, serving as the ionic layer in iontronic pressure sensors. The high porosity (95.4%) of PU-IL composite foam shows a pretty low Young’s modulus of 3.4 kPa and good compressibility. A superhigh maximum sensitivity of 9,280 kPa^−1^ in the pressure regime and a high pressure resolution of 0.125% are observed in this foam-based pressure sensor. The device also exhibits remarkable mechanical stability over 5,000 compression-release or bending-release cycles. Such high porosity of composite structure provides a simple, cost-effective and scalable way to fabricate super sensitive pressure sensor, which has prominent capability in applications of water wave detection, underwater vibration sensing, and mechanical fault monitoring.

**Supplementary Information:**

The online version contains supplementary material available at 10.1007/s40820-021-00770-9.

## Highlights

- High porosity and low modulus of the active layer are found to contribute to high sensitivity in a capacitive pressure sensor.
- Pressure sensor achieves a superhigh sensitivity of 9280 kPa-1 by using polyurethane-ionic liquid foam with a high porosity (95.4%) and a low modulus (3.4 kPa).

**Supplementary Information:**

The online version contains supplementary material available at 10.1007/s40820-021-00770-9.

## Graphical abstract

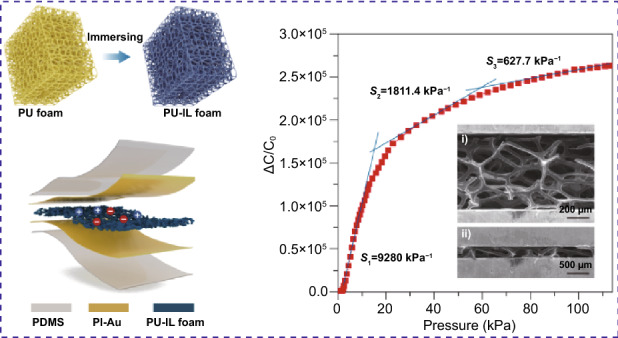

## Introduction

Flexible pressure sensors, a type of devices that can convert mechanical stimuli to electrical signals, have been broadly applied in human–machine interaction, artificial intelligence, and health monitoring [1–5]. According to the sensing mechanisms, pressure sensors can be divided into four common types, including piezoelectric [6], piezoresistive [7], piezocapacitive [8], and triboelectric sensors [9]. Compared with other types, the piezocapacitive sensors show merits of high accuracy, high stability, and simple device construction, thus are widely used to achieve high-performance pressure sensing. However, conventional thickness-governed capacitive sensors present a relatively low sensitivity, this is because the soft dielectric is difficult to compress due to unchanged volume under mechanical deformation, and thus the change of the dielectric thickness is limited. Such a problem can be addressed by introducing diverse microstructures into the dielectric layer to improve its compressibility [10, 11]. Nevertheless, the thickness-governed capacitive sensing devices often fail to reach a high sensitivity of over 10 kPa^−1^ due to the hardening of the microstructures. As a result, engineering the microstructure is not adequate to achieve high sensing performances in a capacitive pressure sensor.

In recent years, ionic conductors have been used to replace the traditional dielectric in capacitive sensors [12, 13]. In this called iontronic sensor, electron double layers (EDLs) that have a fixed charge separation of ~ 1 nm form at the dielectric–electrode interface [14]. This feature leads to two results: First, iontronic sensors present a high unit-area capacitance which is 5–6 orders of magnitude higher than conventional capacitive sensors [15]. Second, thanks to the fixed separation of charges in EDL, the capacitance is mainly determined by the contact area of the dielectric–electrode interface [16], thus creating the possibility to significantly elevate the sensitivity because contact area can be remarkably changed by introducing microstructures at the interface. Based on the EDL sensing mechanism, many capacitive pressure sensors with high sensitivity were reported by programming the contact area change [17]. For example, Park and coworkers employed an incorporate micropatterned pyramidal ionic gel in iontronic capacitive sensors, which exhibited maximum sensitivity up to 41 kPa^−1^ [18]. In 2018, sensitivity of 51.31 kPa^−1^ was achieved by transferring microstructured patterns from *Calathea zebrine* leaf to ionic gel [19]. Recently, Guo and coworkers reported a graded intrafillable architecture in polyvinyl alcohol (PVA)-H_3_PO_4_ ionic gel system. This intrafillable feature enables a significant growth of contact area when loaded with increasing pressure, resulting in an ultrahigh sensitivity of 3,302.9 kPa^−1^ [20]. Therefore, engineering microstructured ionic elastomer has been a considerable strategy in the design of highly sensitive capacitive pressure sensor. The microstructures, however, are often fabricated with sophisticated photolithographic processes, 3D printing, or multiple transfer, which come at a high price. A new selection of ionic materials that can be massively produced and enable high sensitivity is thus required.

Open-cell foams featured as a continuous three-dimensional (3D) network skeleton [21], possess prominent advantages in compressive deformation over surface microstructures. The existence of pores can effectively decrease structural stiffness and improve the compressibility of the material [22, 23]. In addition, many polymers can form foam structures and be made with massive production [24, 25]. The diversity and the cost-effective fabrication of foam materials make foam an ideal selection for flexible pressure sensors. However, it is still challenging to achieve superhigh sensitivity in capacitive pressure sensing system with foam structure for two reasons. First, although the dielectric layer made of foam material may improve the sensitivity to some extent, the thickness-governed sensing mechanism limits the potential sensitivity peak [26, 27]. For another, structural parameters, such as porosity or elastic modulus of foams, have not been received enough attention in iontronic sensors that based on contact area-governed sensing mechanism [28]. Therefore, determining how to combine the advantages of ionic material and foam architecture is an open question.

Herein, we demonstrate that foams of high porosity and low rigidity, when integrated with ionic liquid (IL), achieve superhigh sensitivity up to 9,280 kPa^−1^. An open-cell polyurethane (PU) foam with a porosity higher than 98.8% was selected to serve as the 3D skeleton, then loading IL on the pore walls to create a continuous ionic layer. The PU-IL composite exhibits an extremely low engineering modulus (~ 3.4 kPa) due to the high porosity, and offers minimized initial contact area between the electrode and the ionic layer. The high porosity also promotes structural compressibility, resulting in an ultrahigh pressure resolution of 0.125%. Furthermore, the highly porous foam-based sensor also shows a fast response time of 10 ms, as well as remarkable mechanical stability when subjected to 5,000 compression-release cycles or bending-release cycles. This work provides a simple and effective strategy to massively fabricate super sensitive pressure sensors, and we prove that the devices are potentially useful in applications such as vibration detection underwater and machine failure monitoring.

## Experimental Section

### Finite Element Analysis (FEA)

FEA was performed using the commercial package ABAQUS 6.14. In the PU-IL composite structure, IL layer provides the electrical properties but barely impacts its mechanical properties, and therefore only PU material is considered in this compressive simulation. The PU material was modeled with a compression modulus (*E*) of ~ 6.5 MPa according to experimental measurement. The Poisson’s ratio of PU was set to 0.5. The top polyimide (PI)-Au (*E* ~ 3.5 GPa) electrode was compressed downward while the bottom electrode was fixed. Different porosities were realized by adjusting the thickness of model skeleton thus the architecture remained the same.

### Fabrication of High-porosity Dielectric Layer

IL that we used in this work is 1-Butyl-3-methylimidazolium tetrafluoroborate ([BMIM]BF_4_) which was purchased from Lanzhou Institute of Chemical Physics, China. The open-cell PU foam that fabricated by using gas foaming was provided by Hangzhou Gongshu Meimei Electronic Commerce Co. LTD, China. Flexible and composite ionic foam with high porosity was made by simply immersing the PU foam into IL. The immersing process was implemented at room temperature for 10 min till PU foam was fully loaded with IL. Subsequently, the PU foam was squeezed by a tweezer to remove most of the free IL in its pores, and then placed on dust-free paper to absorb redundant IL. The mass ratio of PU and IL was regulated by weighting. In this work, the mass ratio of IL and PU is ~ 3:1.

### Fabrication of Flexible Electrodes and Sensors

Ion sputter (MC1000) was used to deposit gold (Au) on the surface of flexible polyimide (PI) film to obtain flexible PI-Au electrodes. The parameters of sputtering are 25 mA and 300 s. Before ion sputtering, the PI film was treated for 1 min by using plasma (TS-PL05) to enhance the bonding strength with the Au layer. The PI-Au film was cut by using a CO_2_ laser cutter (WE-6040) into certain sizes such as circular shape of 7 mm in diameter, as well as square shape of 5 × 5 mm^2^ for different tests. The power and speed of the laser cutter were 24 W and 80 mm s^−1^. Silver (Ag) wire was bonded on the side of Au of PI-Au film by silver paste to get final electrode. After that, the PU-IL composite foam was sandwiched between two PI-Au electrodes. Finally, the flexible pressure sensor was packaged using thin PDMS (thickness ~ 50 μm) films. Before packaging, PDMS films and the PI side of top and bottom PI-Au electrodes were treated for 30 s by using plasma to enhance the bonding strength between PDMS-PDMS and PDMS-PI.

### Characterization and Measurements

The morphological analysis of the PU foam and PU-IL composite foam was implemented by using scanning electron microscopy (SEM, TESCAN) operated at 20 kV. Energy-dispersive spectrometry (EDS, OXFORD) was used to analyze the elemental composition of the IL layer. The height distribution of the PU-IL composite foam was explored through a confocal microscope (LSM80C, Carl Zeiss). The compression properties of all samples in this work were tested by using a tensile machine (XLD-20E) at a loading speed of 10 mm min^−1^ at room temperature. The bending cycles were carried out in a home-made testing system. It should be noted that, as an intrinsic characteristic of iontronic devices, the EDL capacitance is highly frequency-dependent. An LCR meter (E4980AL, KEYSIGHT) was used to measure the capacitance of our sensor at a testing frequency of 1 kHz to reveal the sensing ability. In blower fault monitoring, in order to meet the requirement of sampling frequency of LCR meter which should be higher than twice the blower signal frequency (Nyquist’s sampling theorem), the testing frequency was set to 1 MHz to collect the vibration signal of blower. The porosity (*P*) of various foams was determined by the weight, according to Eq. (1):

1 $$P = \frac{{{{\left( {m_{w} - m_{d} } \right)} \mathord{\left/ {\vphantom {{\left( {m_{w} - m_{d} } \right)} {\rho_{w} }}} \right. \kern-\nulldelimiterspace} {\rho_{w} }}}}{{{{\left( {m_{w} - m_{d} } \right)} \mathord{\left/ {\vphantom {{\left( {m_{w} - m_{d} } \right)} {\rho_{w} + {{m_{d} } \mathord{\left/ {\vphantom {{m_{d} } {\rho_{d} }}} \right. \kern-\nulldelimiterspace} {\rho_{d} }}}}} \right. \kern-\nulldelimiterspace} {\rho_{w} + {{m_{d} } \mathord{\left/ {\vphantom {{m_{d} } {\rho_{d} }}} \right. \kern-\nulldelimiterspace} {\rho_{d} }}}}}} \times 100\%$$

where *m*_*d*_ is the mass of initial foam, *m*_*w*_ expresses the mass of the foam loading with liquid, *ρ*_*d*_ and *ρ*_*w*_ are the densities of the foam raw material and liquid, respectively.

## Results and Discussion

### Sensing Mechanism and Finite Element Analysis

For capacitive pressure sensors, sensitivity (*S*) can be expressed as $S = \delta {{\left( {{{\Delta C} \mathord{\left/ {\vphantom {{\Delta C} {C_{0} }}} \right. \kern-\nulldelimiterspace} {C_{0} }}} \right)} \mathord{\left/ {\vphantom {{\left( {{{\Delta C} \mathord{\left/ {\vphantom {{\Delta C} {C_{0} }}} \right. \kern-\nulldelimiterspace} {C_{0} }}} \right)} {\delta P}}} \right. \kern-\nulldelimiterspace} {\delta P}}$, where *C*_0_ is initial capacitance before applying pressure ($P$), and ∆*C* = *C*−*C*_0_ presents the capacitance change upon loading. In an iontronic sensor, the variation of EDL capacitance is the key to improve sensitivity. According to the classic EDL model, the induced capacitance of an interfacial EDL can be expressed as the following format [29]:

2 $$C_{{{\text{EDL}}}} = {\text{UAC }}\cdot \, A$$

where UAC is unit area capacitance that can reach a few μF·cm^−2^ due to the nanoscale distance between the positive and negative charges at the EDL interface; *A* is the contact area between the ionic layer and electrode. The UAC is mainly determined by material properties such as ion type and density, and thus can be regarded as a constant in a given system. Therefore, the signal is generated when the contact area is changed by applying a pressure. According to the Persson contact theory for a randomly rough surface, the normalized contact area can be expressed as [30]:

3 $$\frac{\Delta A}{{A_{0} }} = \frac{{A - A_{0} }}{{A_{0} }} = \psi \frac{P}{E}$$

where *ψ* is a geometric parameter that depends on the surface morphology, *E* is the effective elastic modulus of the structure, *A*_0_ is initial contact area, and *∆A* = *A*−*A*_0_ is the change of contact area under external loading. Therefore, *A*_0_ and *E* should be as low as possible to pursue a larger *∆A* or higher sensitivity. A small *A*_0_ can be achieved by using a highly porous architecture to increase air-occupied space. In addition, engineering modulus *E* of the porous structure will also be significantly decreased compared with their bulk counterpart according to the Gibson and Ashbury theory [31]:

4 $$E = E_{0} D\left( {1 - V_{P} } \right)^{n}$$

where *E*_0_ is the elastic modulus of a bulk material, *D* and *n* are geometric constants related to the specific microstructure. *D* ≈ 1 and 1 ≤ *n* ≤ 4 are often used for high-porosity materials, and here *V*_*p*_ is the porosity. As such, highly porous structures will exhibit a low *E* and a small *A*_0_, which is desired to achieve high sensitivity in iontronic pressure sensors.

**Fig. 1:**
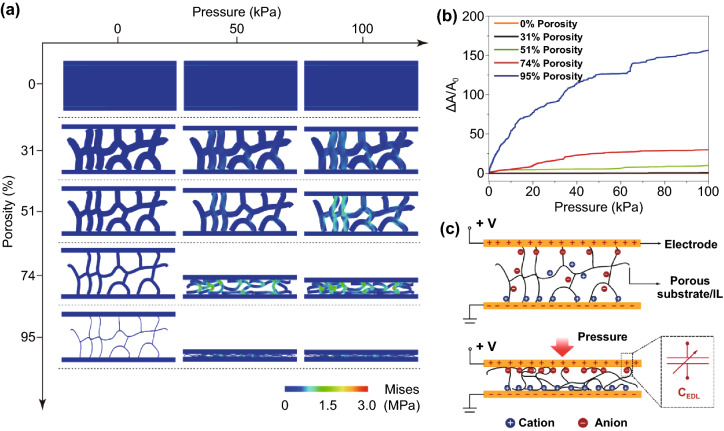
Deformation of porous materials with different porosities upon compression, and the sensing mechanism. **a** Simulated stress distribution of architectures with different porosities of 0%, 31%, 51%, 74% and 95%. **b** Contact area variation between the dielectric layer with different porosities and electrodes in a sensing range of 0–100 kPa. **c** Schematic illustration for sensing mechanism of the foam-based iontronic pressure sensor

We investigate the compression process of porous structures with different porosities and elastic moduli to elucidate the deformation mechanism. By performing FEA, schematics of different compressive stages are shown in Fig. 1a. Two important properties are studied: compressibility, and normalized change in contact area, which determine the sensing properties of the iontronic sensors. Nonporous soft materials are often incompressible, say, volume of the material maintains constant to allow no space for internal deformation. Therefore, such materials exhibit a high resistance to external pressure and are not suitable for pressure sensing. By contrast, when interconnected micropores are introduced to a soft material, compressibility will be significantly improved because air in the micropores can be squeezed out and enable internal deformation. We can see from the simulation that compressibility increases significantly as the porosity increases.

When a rigid plate is placed on top of the porous material, the interfacial contact area will change upon loading. Figure 1b shows the normalized change of contact area (∆*A*/*A*_0_) as a function of pressure for the soft material with different porosities. The simulation indicates that under a given pressure, ∆*A*/*A*_0_ increases as porosity increases. The initial contact area *A*_0_ is determined by the number and thickness of ligaments that are in contact with the electrode, which is inversely associated with porosity. As a result, high porosity is preferred for improving sensitivity. Note that in a real foam-electrode interface, only a few ligaments are in contact with the electrode before loading, and thus the true *A*_0_ value is smaller than the simulated value. As such, the practical ∆*A/A*_0_ value will be much larger than the simulated result. The sensing mechanism is illustrated in Fig. 1c, which shows that massive positive and negative ion pairs are distributed at the foam–electrode interface, and the signal amplitude of the interface is determined by the contact area. Therefore, the capacitive signal can reflect the contact area and the pressure applied to the device.

**Fig. 2:**
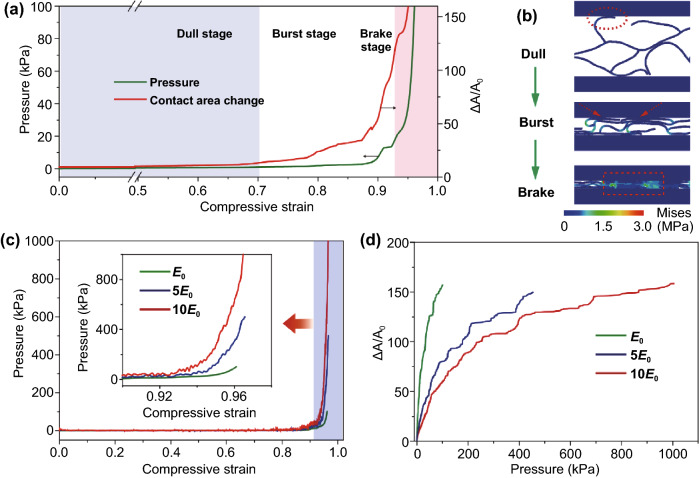
FEA simulation on the compressibility and change in contact area of the foam. **a** The evolutions of pressure and normalized contact area in compressive deformation process of a foam with 95% porosity. **b** Contact forms between ligaments of the foam and electrodes in different compressive stages. **c** Stress–compressive strain curves of the foam with 95% porosity and different material moduli (*E* = *E*_0_, 5*E*_0_, 10*E*_0_). **d** Contact area variation between the dielectric layers with different material moduli and electrodes as a function of applied pressure

According to the evolution of key factors along with the increasing of compressive strain in simulation, the entire deformation process can be divided into a dull stage, a burst stage, and a brake stage, as shown in Fig. 2a. In the dull stage, although it crosses a wide compressive strain range, both the loaded pressure and contact area maintain at a very low level due to the high porosity, and a few ligaments are in point contact with the top plate (see red circle in Fig. 2b) to generate a small contact area. When coming into burst stage, the mediate layer has been highly compressed and the limited vacant spaces force the ligaments to be parallel to the top plate, which substantially contribute to the overall contact area, as marked by the red arrows in Fig. 2b. Importantly, there are many residual air pores to separate neighboring ligaments, thus the structures still exhibit high compressibility with low resistance to external loading, different from its nonporous counterpart. As a result, the contact area increases rapidly at this stage, while the pressure grows slowly, resulting in ultrahigh sensitivity. After nearly all the air is squeezed out from the porous part, the compressibility decreases and the deformation transits to the brake stage, as shown in Fig. 2b with a red dashed box. In this last stage, the structure is densified at some parts, thus the compressibility decreases and causes extreme increase in pressure at a narrow strain range. However, the contact area also increases before fully saturated, maintaining the sensitivity at a high value.

Besides porosity, the elastic modulus of the material also affects the sensing properties of the device. This implication is supported by simulated results on the deformation of highly porous materials (95% porosity) with different elastic moduli of *E*_0_, 5*E*_0,_ and 10*E*_0_ (*E*_0_ = 6.5 MPa), and the results are shown in Fig. S1. When the porosity of the architecture and applied pressure are all fixed, the compressibility is largely determined by the elastic modulus of the material. As shown in Fig. 2c, when the elastic modulus is set to 10*E*_0_, the porous structure needs a pressure higher than 1,000 kPa to achieve a ~ 96% compressive strain. However, in the case of *E* = *E*_0_, 100 kPa adequate to realize the same compressive strain. In addition, the normalized contact area ∆*A/A*_0_ is also inversely associated with the elastic modulus of the material, as shown in Fig. 2d. Based on the FEA results, we can clearly suggest that the low modulus and high porosity are critical factors to produce a sensitive response to pressure.

### Properties of the PU-IL Composite Foam

**Fig. 3:**
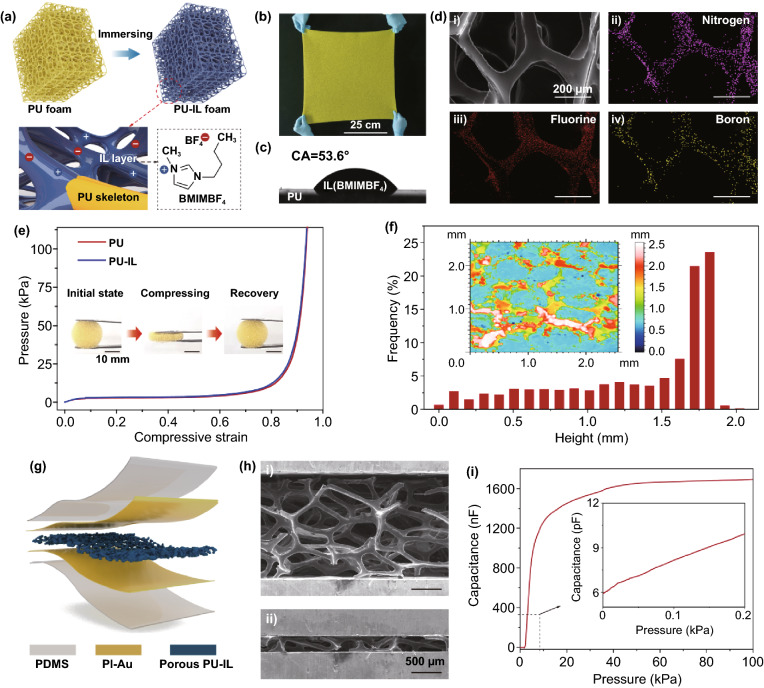
PU-IL composite foam, flexible capacitive-type pressure sensor and its sensing property. **a** Schematic of a PU-IL composite foam with IL on the surface of PU skeleton. **b** A PU-IL composite foam with a dimension of 50 cm × 50 cm × 2 mm. **c** Contact angle of IL on the surface of PU plate. **d** Elemental mapping of nitrogen, fluorine and boron of the PU-IL composite foam. **e** Stress–strain curves of PU and PU-IL composite foams under compression. **f** Height distribution of the PU-IL composite foam. **g** Schematic of a capacitive-type pressure sensor that consists of two PI-Au electrodes, a PU-IL composite foam and a PDMS sealing layer. **h** SEM images of the PU-IL foam before and after compression. **i** Capacitance as a function of pressure of the sensor using our PU-IL composite foam

Based on the mechanism analysis above, an open-cell PU foam with ultrahigh porosity up to 98.8% was selected as the porous elastomer. PU is a soft material with a low elastic modulus of ~ 6.5 MPa (Fig. S2). Thanks to the high porosity, the modulus of the PU foam is reduced to ~ 3.4 kPa. Next, the highly porous PU foam was immersed into IL (1-butyl-3-methylimidazolium tetrafluoroborate, BMIMBF_4_). Followed by a compression step to squeeze free IL out, a thin layer of IL was formed on the PU skeletons, as illustrated in the schematic diagram (Fig. 3a) and SEM observation (Fig. S3). After loading the IL, the composite foam still maintains a high porosity (95.4%). Such a PU-IL composite foam can be easily made at a large area and low cost. In Fig. 3b, we show a large area sample with dimensions of 50 cm × 50 cm × 2 mm. The contact angle of IL on the surface of PU plate is 53.6°, which proves the IL has a good wettability for PU material, as shown in Fig. 3c. It is worth noting that the thin IL layer can be stably distributed on the PU microstructure surface due to its negligible volatility and low interfacial tension (3.15 Mn m^−1^) with PU. In addition, the interaction of IL layer with PU matrix is enhanced due to the polarization effect between the high electronegative oxygen (O) atoms from ether group (C–O–C) and BMIM^+^ cations [32]. Fourier transform infrared spectroscopy (FTIR) spectra results in Fig. S4 confirmed this point, the absorption peaks of stretching vibration of C–O–C in PU-IL composite foam (1092 cm^−1^) are red-shifted relative to that in pure PU foam (1071 cm^−1^), indicating an interaction intensification between PU matrix and IL layer. Furthermore, the existence of thin IL layer on the PU micro-surface is proven by EDS results. Because [BMIM]BF_4_ in the IL is the only source of fluorine (F) and boron (B) elements in our composite, the EDS mapping images of the F and B clearly evidence that the IL is uniformly distributed on the walls of PU skeleton (Fig. 3d). These experimental results clearly indicate that the IL is relatively stable in the PU-IL composite foam.

In the PU-IL foam, the mechanical properties are largely determined by the PU foam while the electrical properties are determined by the IL layer. As shown in Fig. 3e, the well overlapped stress–strain curves of PU and PU-IL foams prove that IL has little contribution to the stiffness. In addition, the PU-IL foam can perfectly return to its initial state while loading is removed (inset of Fig. 3e), showing an excellent recovery property. Furthermore, the surface roughness of the PU-IL foam is important because the contact area between the foam and the electrode determines the capacitive signal. Figure 3f shows a statistic height distribution of the PU-IL foam with thickness of ~ 2 mm measured by using a confocal microscope. The height values (0–2 mm) represent the distance between the top surface of porous foam and bottom substrate. The three-dimensional skeletons and pores in PU-IL foam result in a broad height range, indicating a large surface roughness. In the initial state, only the highest ligaments (height ~ 2 mm) that have the largest height value, corresponding to the brightest areas in color map (inset of Fig. 3f), contact to the electrode, leading to a significantly small initial contact area A_0_, which is helpful in promoting the sensitivity of the device, as discussed in Sect. 3.1.

An iontronic capacitive-type pressure sensor was fabricated by sandwiching the open-cell PU-IL foam between two PI-Au electrodes. The sensor was then sealed by using a thin PDMS film and the schematic is shown in Fig. 3g. From the SEM images of the pressure sensor before compression (Fig. 3h), we can clearly see that only a few PU-IL fulcrums touch the electrodes at the initial state owing to the high porosity. When an external force is applied, the porous PU-IL layer is deformed and the air in the interconnected pores is squeezed out. The corresponding capacitance–pressure curve is shown in Fig. 3i. At the unloading state (0 kPa), the small contact area results in a minimal initial capacitance of ~ 6 pF, for which the EDL capacitance has little contribution, and this can be verified by the fact that the signal amplitude has little relation of testing frequency (Fig. S5a). Upon loading, the electrode–foam contact area significantly increases, and the capacitance value acutely increases to ~ 1.76 μF, a 6 orders of magnitude improvement compared with the initial value. It should be pointed out that the electrical properties of ion conductors are related to the testing frequency [33], and thus the EDL capacitance in iontronic sensors is highly frequency-dependent (Fig. S5b). The results demonstrate that the highly porous PU foam that loads with a thin IL layer is an ideal selection of ionic material to improve the capacitance–pressure response in iontronic pressure sensors.

### Sensing Properties of the Ionic Foam-based Pressure Sensor

**Fig. 4:**
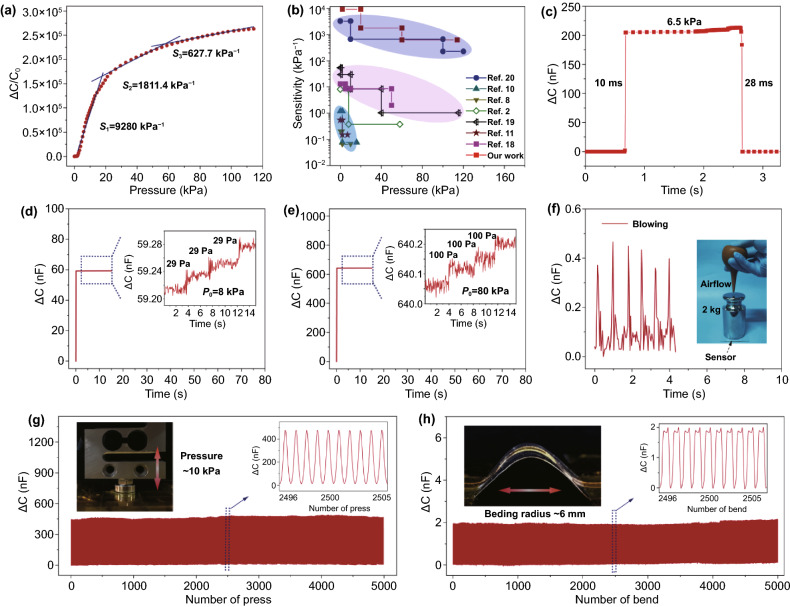
Sensing performances of the PU-IL foam-based capacitive pressure sensor. **a** Sensitivity of the sensor at pressure range up to 114 kPa. **b** Comparison of the sensitivity of our device with previously reported results. **c** Response time of the pressure sensor. Detection of weak pressure at preload pressure of 8 kPa (panel **d**) and 80 kPa (panel **e**). **f** Capacitance response of our sensor to airflow under a basic pressure of 127 kPa from 2 kg weight. **g** Compression-release stability over 5000 cycles under a peak pressure of 10 kPa. **h** Bend-release stability over 5000 cycles to a bending radius of ~ 6 mm

Sensitivity is a crucial parameter for pressure sensors that presents the ability to detect the changes in external pressure. The capacitance–pressure curve of our PU-IL foam-based pressure sensor is shown in Fig. 4a. After a short dull stage at the beginning (< 2 kPa), the sensitivity booms to an ultrahigh value of 9,280 kPa^−1^ in the pressure range of 2–20 kPa. Subsequently, the sensitivity decreases but still stays at high levels of 1811.4 kPa^−1^ in 20–60 kPa and 627.7 kPa^−1^ in 60–114 kPa. To the best of our knowledge, the maximum sensitivity of our sensor is higher than that of existing traditional parallel-plate and EDL-based capacitive-type sensors reported in literature (Fig. 4b). In the dull stage, the electrodes contact with a few PU tips of the foam surface, and such structures buckles while no significant change in contact area occurs because the side wall of the PU ligaments is not in contact with the electrodes yet. The dull stage can be effectively eliminated by introducing a pre-pressure during device encapsulation. As the pressure further increases, the electrode starts to contact with the side wall of the ligaments, and thus a significant change in contact area or capacitive signal occurs. Because of the high porosity and low modulus of PU foam, a high sensitivity is achieved. The contact starts to get saturated as pressure increases to higher than 100 kPa, at which the foam is remarkably compressed. The whole deformation process of PU-IL foam is presented in Movie S1. It is worth pointing out that the working range of the ionic foam-based pressure sensor can be readily scaled by changing the elastic modulus of the material. For example, when we use the melamine foam (elastic modulus ~ 24.5 kPa, 7 times that of the PU foam) that has a porosity of ~ 98.8% to replace the PU foam, the sensing range broadens to ~ 700 kPa, 7 times that of the PU-IL foam-based sensor, while the maximal sensitivity drops to 1,334.4 kPa^−1^, which is about 1/7 that of the PU-IL foam-based sensor (Fig. S6). To reach the same normalized change of contact area ∆*A*/*A*_0_, one needs to apply a greater pressure to deform stiffer materials according to the Persson contact theory (see Sect. 3.1), which will result in a relatively low sensitivity while simultaneously broadening the working range. The result suggests that the foam substrate in our strategy can be selected flexibly to better meet the practical application requirements of different sensitivities and pressure ranges.

Furthermore, the response speed of the pressure sensor is investigated by loading and unloading a weight that causes 6.5 kPa pressure on the sensor. The ascending and descending time of the capacitance signal are 10 and 28 ms (Fig. 4c), respectively, revealing a much shorter response time than that of human skin [34]. This fast response speed lies in the elastic feature of the PU skeleton, although it has been partially slowed down due to viscoelastic nature of the IL that can be evidenced by the small hysteresis in our sensor (Fig. S7). In addition, the ultra-sensitive response allows for high-pressure resolution. We tested the capability of the sensor to resolve tiny pressure changes at base pressures of 8 and 80 kPa which represent a low and a high pressure. We placed light-weight plastic blocks with weights of 74.2 mg (~ 29 Pa) at an 8 kPa base pressure, and 255.3 mg (~ 100 Pa) at an 80 kPa base pressure, and clear stepped capacitance increments were observed, as shown in Fig. 4d, e. When the sensor is in the ultrahigh sensitivity region under a base pressure of 3.25 kPa, even a tiny pressure change (5.2 Pa) leads to a considerable change in signal amplitude (Fig. S8). This sensor can also sense slight pressure changes at high pressures. As shown in Fig. 4f, when we placed a metal weight (2 kg or ~ 127 kPa) on a circle-shaped sensor with a radius of 7 mm, the sensor showed sharp capacitance response to the mild air flow generated by squeezing a rubbery bulb.

For an ideal flexible pressure sensor, long-term working stability is one of the most desired properties. As shown in Fig. 4g, h, our sensor exhibits stable response over either 5,000 compression-release cycles with a peak pressure of 10 kPa, or 5,000 bend-release cycles with a bending radius of ~ 6 mm. The amplitude and waveform of capacitance signal remains unchanged during the compression-release and bend-release cycling tests, as shown in the insets of Fig. 4g, h. EDS results illustrate that the IL layer is well preserved on the surface of the PU skeleton even after 5,000 compression-release cycles (Fig. S9), which further verifies the working stability of the sensor. In addition, as shown in Fig. S10, the sensitivity of our sensor just has a slight decrease after 5000 compression cycles and still maintains a superhigh value of 9,193 kPa^−1^. Besides, the pressure sensor also shows an adaptability to the environment with different humidity and temperatures (Fig. S11). Thanks to the complete sealing, the change in relative humidity of the air does not affect the capacitance. While the signal intensity grows as the temperature increases due to the enhanced mobility of ions under elevated temperature, our sensor shows a lower capacitance signal drift compared with other iontronic sensors due to heat insulation effect of foam structure [20, 35].

### Application of the Sensor for Water Wave Detection and Engineering Vibration Monitoring

**Fig. 5:**
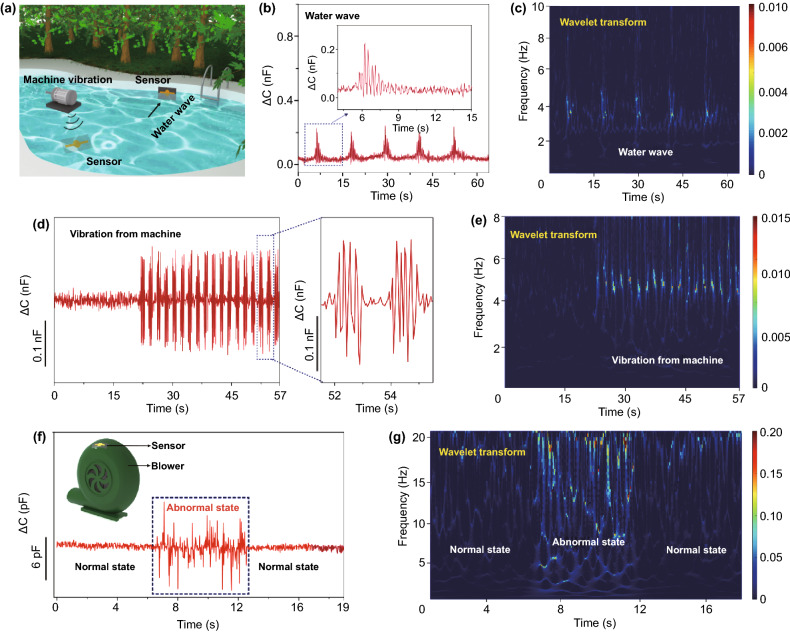
Applications of the PU-IL foam-based pressure sensor. **a** Schematic illustration of the application scenarios of the sensor. **b** Waveform of water waves in time domain detected by using our pressure sensor, inset shows the waveform of a single wave envelope. **c** Frequency-time window of water wave signals obtained by using wavelet transform. **d** Vibration signals of machine underwater detected by the sensor. **e** Frequency-time window of vibration signals from a machine underwater. **f** Capacitance signals of a sensor attached to a blower at normal and abnormal working conditions. **g** Frequency-time window of the blower at normal and abnormal working conditions

The superhigh sensitivity allows the sensor to detect tiny mechanical stimuli. Here we demonstrate the ability of the sensor to measure water wave signals, and the application scenarios are shown in Fig. 5a. We threw a marine ball (~ 5.9 g) on water to generate water waves. The wave propagates from the wave source and hits the side wall of the pool. A sensor was attached to the pool wall at the water–air interface to detect the pressure caused by the waves, together with the frequency and intensity information over time. As shown in Fig. 5b and its inset, the detected capacitance signal consists of a few sets of repeated waves with damped magnitude. Through wavelet transform that provides a frequency-time window, one can see a distinct and periodic frequency signal evolution, as shown in Fig. 5c. For a single set of waves, both the frequency and intensity will experience a damped decrease due to the energy decay of water wave with time. In addition, sensor was also placed at the bottom of pool with a depth of 20 cm (water pressure ≈ 2 kPa) to detect the vibration signal of a motor underwater (Fig. 5d). The water body vibrates when the machine is working, and waves with characteristic vibration frequency can be detected using the sensor (Fig. 5e).

Another possible application is fault diagnosis of machines. As illustrated in Fig. 5f, we attached the sensor on the surface of a blower and collected the dynamic capacitance outputs. The testing frequency was set to 1 MHz in order to match the mechanical vibration frequency. To simulate the abnormal working state, we blocked the air inlet of blower, while air inlet was open as the normal working state. We can judge whether the machine works normally through the analysis of the signal amplitude in the time domain. The malfunction of the machine is often accompanied with unusual noise, which is converted to irregular capacitance outputs by our ultrasensitive sensor. Furthermore, wavelet transformation of the capacitive signal helps detect the anomalous state not only from the frequency information but also the intensity of the signal (Fig. 5g). These experimental results indicate that the PU-IL foam-based pressure sensor has extraordinary potentials to be used in the machine fault diagnosis due to its capability of sensing tiny vibration signals.

## Conclusions

In summary, we successfully achieved a PU-IL foam-based pressure sensor with an ultrahigh sensitivity of 9,280 kPa^−1^, which benefits from its high porosity and low elastic modulus. In the composite foam structure, the PU 3D foam serves as an elastic structure, while the IL layer determines the electrical properties to form interfacial EDL capacitance. Thanks to the low interfacial tension and interaction intensification, the IL layer can uniformly distribute on the PU surface to form stable ionic foam. The highly sensitive pressure sensor not only has a high-pressure resolution but also shows a good mechanical stability. Such an elastic foam-based iontronic pressure sensor exhibits great potentials in wave fluctuation and vibration detection underwater, as well as the diagnosis of machine health. This work opens a door for low-cost and massive fabrication of iontronic pressure sensors with superhigh sensitivity.

## Supplementary Information

Below is the link to the electronic supplementary material.

Supplementary file1 (MP4 7695 KB)

Supplementary file2 (PDF 946 KB)
