# Supplementary material for: High-Porosity Foam-Based Iontronic Pressure Sensor with Superhigh Sensitivity of 9280 kPa−1
Source: Nanomicro Lett. 2021 Dec 9;14:21. doi: 10.1007/s40820-021-00770-9 (PMC8660951; doi:10.1007/s40820-021-00770-9)
Supplement: Supplementary file 2 — Supplementary file2 (PDF 946 KB) [file 40820_2021_770_MOESM2_ESM.pdf]

Supporting Information for

High-porosity Foam-based Iontronic Pressure Sensor with Superhigh Sensitivity of 9280 kPa^{-1}

Qingxian Liu[1, 2, 3, 7], Yuan Liu[6], Junli Shi[2, 7], Zhiguang Liu[2, 5, *], Quan Wang[3, 4, *], Chuan Fei Guo[2, 7, *]

[1]School of Astronautics, Harbin Institute of Technology, Harbin, Heilongjiang 150001, P. R. China

[2]Department of Materials Science and Engineering, Southern University of Science and Technology, Shenzhen 518055, P. R. China

[3]Department of Mechanics and Aerospace Engineering, Southern University of Science and Technology, Shenzhen, Guangdong 518055, P. R. China

[4]Department of Civil and Environmental Engineering, Shantou University, Shantou, Guangdong 515063, P. R. China

[5]Department of Mechanical Engineering, Massachusetts Institute of Technology, Cambridge, MA 02139, USA

[6]Department of Physics and TcSUH, University of Houston, Houston, TX 77204. Current address: 320 Crescent Village Circle Unit 1413, San Jose, CA 95134, USA

[7]Guangdong Provincial Key Laboratory of Functional Oxide Materials and Devices, Southern University of Science and Technology, Shenzhen 518055, Guangdong, P. R. China

*Corresponding authors. E-mail: liuzg@sustech.edu.cn (Zhiguang Liu); wangq@sustech.edu.cn (Quan Wang); guocf@sustech.edu.cn (Chuan Fei Guo)

Supplementary Figures

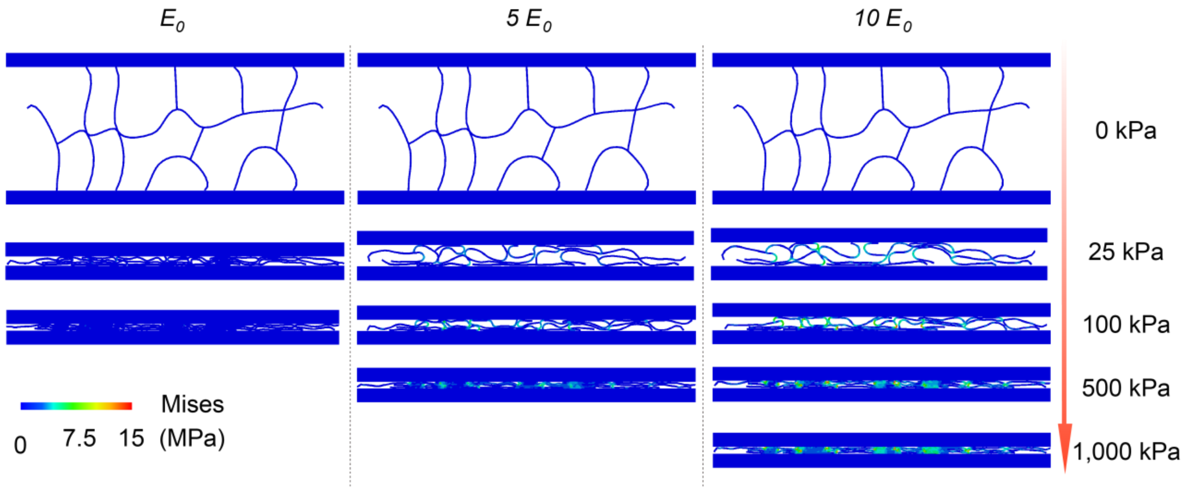

Fig. S1 Stress distribution of FEA results for foams (95% porosity) with different moduli of E_0, $5E_0$ and $10E_0$, where E_0 = 6.5 Mpa

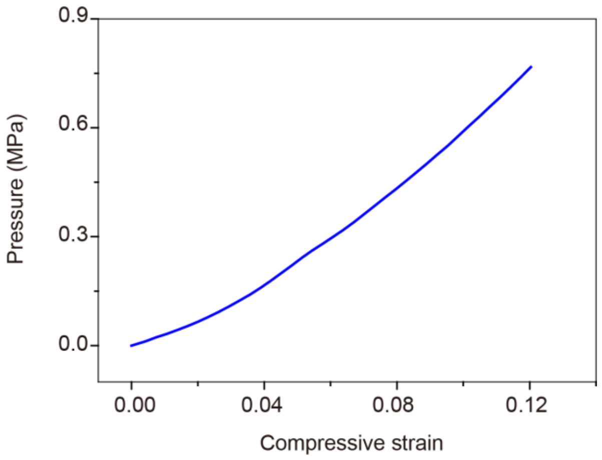

Fig. S2 Compression stress-strain curve of non-porous PU plate showing the modulus is ~6.5 MPa that evaluated by computing slope curve

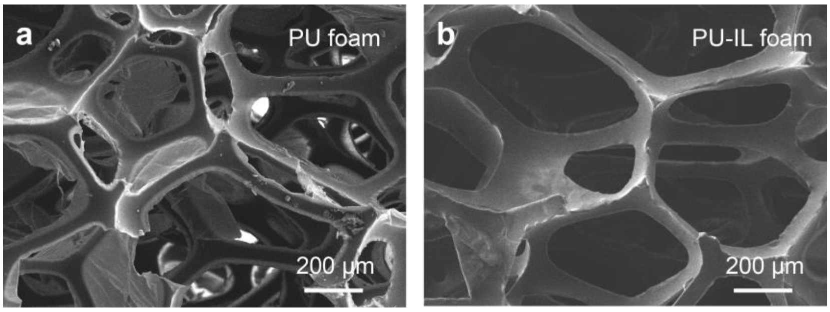

Fig. S3 Scanning electron microscope (SEM) images of initial PU foam (**a**) and the PU-IL composite foam (**b**) It is clearly shown that the IL is just on the pore walls rather than in the pores, while PU-IL composite foam still maintains high porosity (95.4%). The mass ratio of the PU skeleton and IL in the composite foam is about 1:3

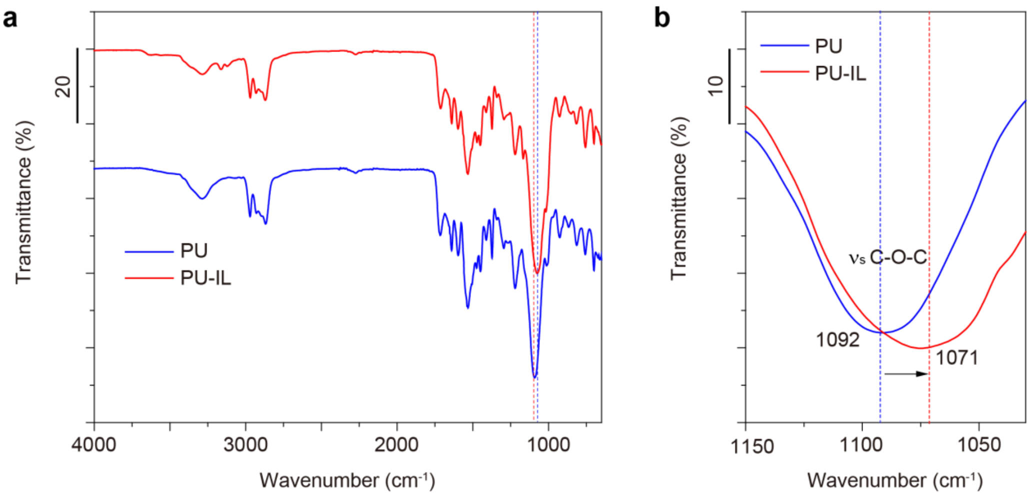

Fig. S4 (**a**) FTIR spectra of PU foam and PU-IL ($BMIMBF_4$) composite foam. (**b**) The partial amplified FTIR spectra shows an ether (C-O-C) band shifting to lower wavenumber from 1092 cm^{-1} to 1071 cm^{-1}. The FTIR results suggest that the cations (imidazolium) of $BMIMBF_4$ interacte with ether group to some extent, indicating an interaction intensification

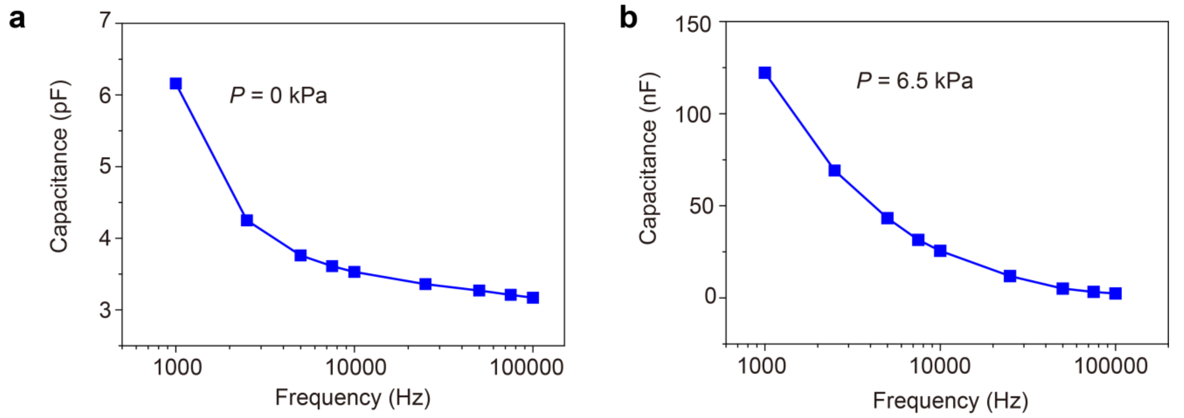

Fig. S5 (**a**) Capacitance-frequency curve of a PU-IL foam-based pressure sensor at unloading state (0 kPa). (**b**) Capacitance-frequency curve of a PU-IL foam-based sensing device under a pressure of 6.5 kPa

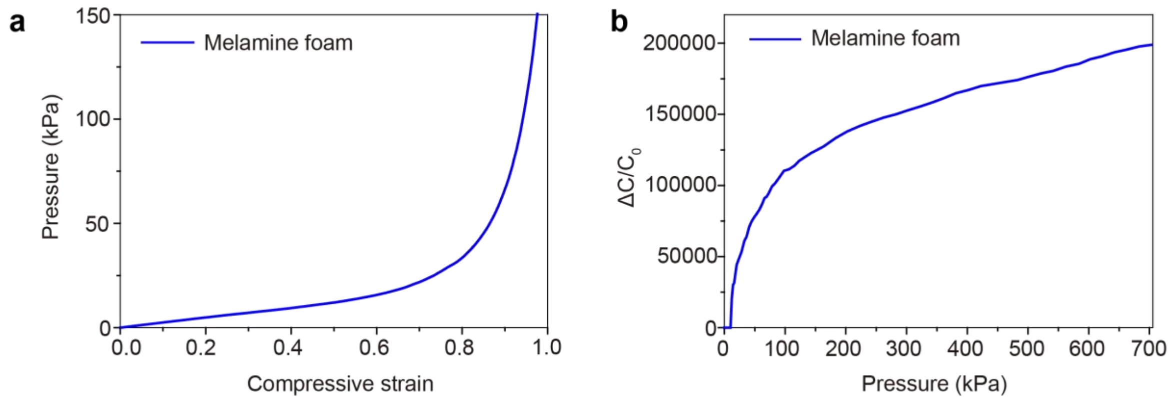

Fig. S6 The melamine foam with 98.8% porosity was selected to load IL. (**a**) Compression stress-strain curve of melamine-IL foam, compression modulus of which is 24.5 kPa evaluated with in the linear strain range (0-0.6). (**b**) Sensitivity of the melamine-IL foam-based pressure sensor under various pressure range. The sensitivity value is 1344.4 kPa^{-1} at 10-100 kPa, 280.4 kPa^{-1} at 100-200 kPa and 121.2 kPa^{-1} at 200-700 kPa

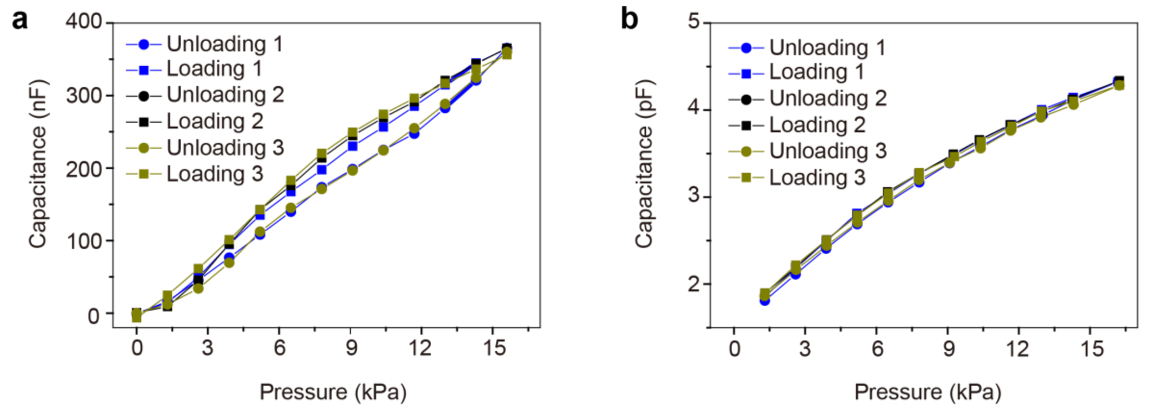

Fig. S7 Hysteresis of (**a**) PU-IL composite foam-based and (**b**) PU foam-based pressure sensors in three loading-unloading cycles

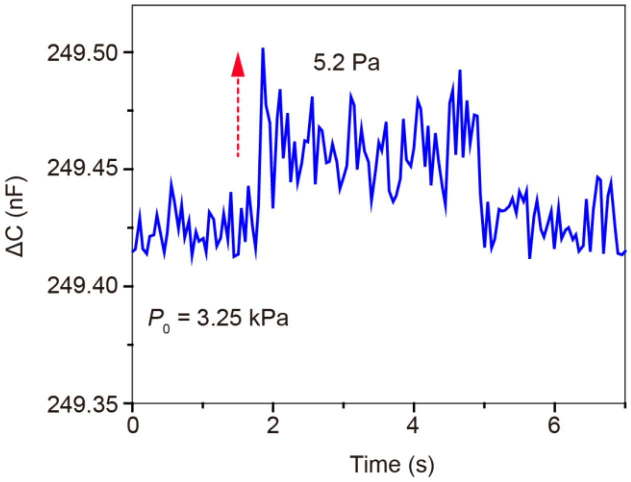

Fig. S8 Resolving a tiny pressure change of 5.2 Pa under a referent pressure of 3.25 kPa

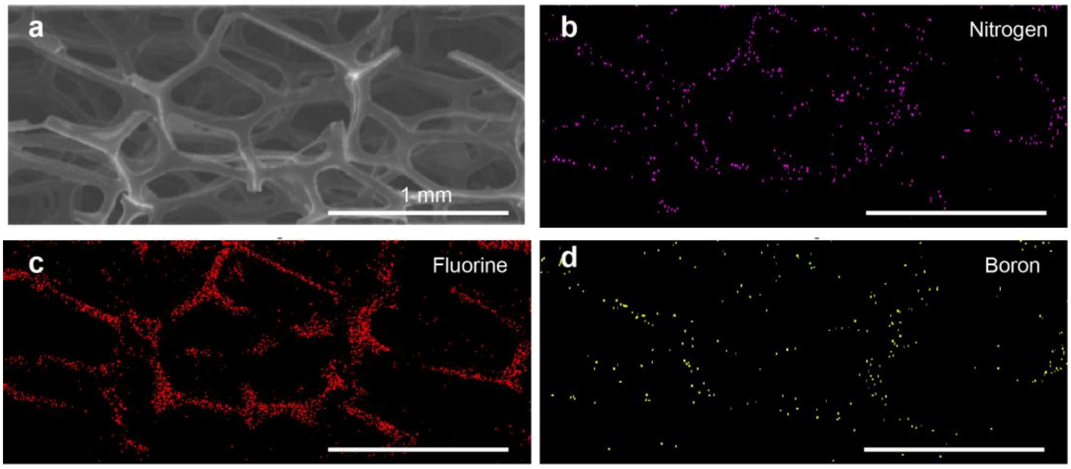

Fig. S9 EDS images of the PU-IL foam (**a**) after 5000 compression cycles at a pressure of ~10 kPa. From the distribution of elements including nitrogen (**b**), fluorine (**c**) and boron (**d**), we can further verify that the IL always attaches on the surface of the PU skeleton and maintains structural stability even during a long period of mechanical compression

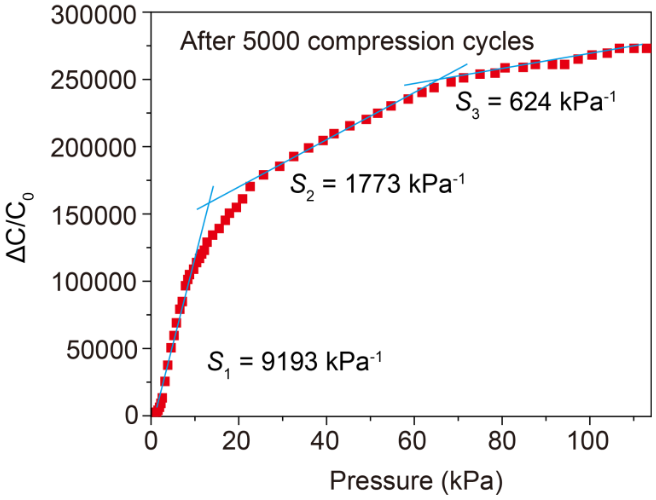

Fig. S10 Sensitivity of the PU-IL composite foam-based sensor after 5000 compression cycles

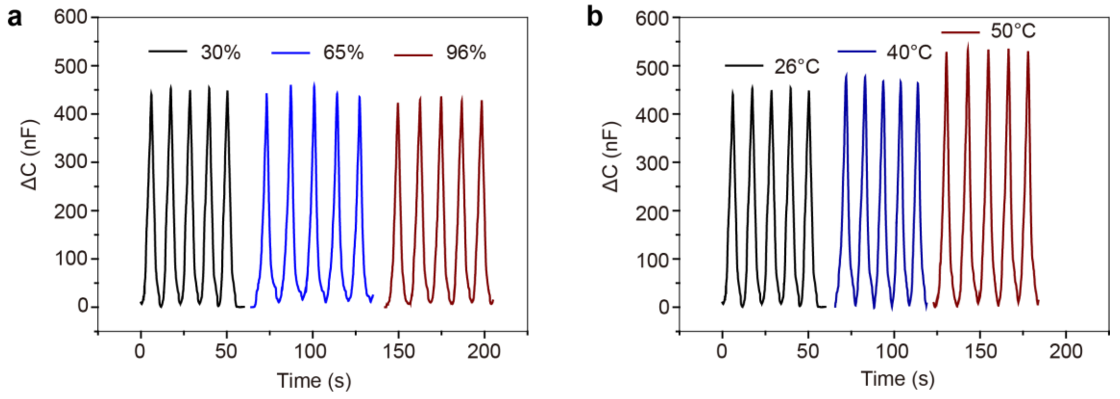

Fig. S11 Capacitance response of our sensor under different (**a**) humidity and (**b**) temperature conditions
